# The Effect of Hyperhomocysteinemia on Motor Symptoms, Cognitive Status, and Vascular Risk in Patients with Parkinson's Disease

**DOI:** 10.1155/2016/1589747

**Published:** 2016-08-25

**Authors:** Bilge Kocer, Hayat Guven, Isik Conkbayir, Selim Selcuk Comoglu, Sennur Delibas

**Affiliations:** ^1^Department of Neurology, Diskapi Yildirim Beyazit Training and Research Hospital, 06110 Ankara, Turkey; ^2^Department of Radiology, Diskapi Yildirim Beyazit Training and Research Hospital, 06110 Ankara, Turkey

## Abstract

Factors related with hyperhomocysteinemia (HHcy) and the impact of HHcy in Parkinson's disease (PD) are not well understood. We investigated the factors associated with increased levels of homocysteine (Hcy) and the relationship between HHcy and motor symptoms, cognitive status, and vascular risk in patients with Parkinson's disease. Among 60 patients (29 males, 48.3%) with PD, the stage of the disease, the severity of clinical symptoms, and the patients' cognitive status were measured using a modified Hoehn and Yahr Staging Scale (mHY), Unified Parkinson's Disease Rating Scale (UPDRS) II and III, and Mini-Mental State Examination (MMSE), respectively. Patients were also noted for having dyskinesia and hallucinations. Serum vitamin B12, folic acid, and plasma Hcy levels were measured. Furthermore, the presence of vascular risk factors was recorded. Finally, we investigated carotid artery intima-media thickening and stenosis using colour Doppler ultrasonography as well as the presence of ischemic lesions using brain imaging techniques. Plasma Hcy levels were higher with advanced age and in males. In addition, there was an inverse relationship between Hcy and vitamin B12 levels. There was no correlation between HHcy and the stage of the disease, severity of motor symptoms, cognitive status as assessed by the MMSE, vascular risk factors, carotid artery atherosclerotic findings, and ischemic brain lesions. Plasma Hcy levels may rise due to several factors in PD. However, the resulting HHcy has no significant effect on the clinical picture in terms of motor features, cognitive status, and vascular diseases.

## 1. Introduction

Hyperhomocysteinemia (HHcy) is an established risk factor for cardiovascular, cerebrovascular, and peripheral vascular diseases [1–3]. In Parkinson's disease (PD) patients undergoing levodopa (LD) therapy, plasma homocysteine (Hcy) levels are elevated as a result of the transmethylation of LD via catechol O-methyl transferase (COMT) [4, 5]. The effect of this HHcy on vascular diseases in PD patients is unclear; HHcy could potentially cause vascular pathologies or the worsening of motor features.

Experimental studies have demonstrated that Hcy can be neurotoxic and excitotoxic to the substantia nigra. Furthermore, Hcy may be associated with dyskinesias, which is an indicator of possible neurodegeneration due to the disruption of the balance of striatal activity [6, 7].

The prevalence of neuropsychiatric symptoms, such as depression and dementia, is increased in PD [8, 9]. Among the elderly, HHcy is a well-known risk factor for dementia [10]. HHcy has been proposed to also be a risk factor for the neuropsychiatric disorders, cognitive deterioration, dementia, and depression that are seen in PD, but some studies have not confirmed these results [11–15].

Therefore, we examined the relationship between plasma Hcy levels and the severity of PD-related motor features and cognitive status. We also determined the risk of vascular disease in PD patients by using vascular risk factors, previous vascular diseases, atherosclerotic findings detected by carotid artery colour Doppler ultrasonography, and ischemic changes in brain imaging studies.

## 2. Materials and Methods

### 2.1. Study Design

This prospective study included 60 patients (29 males, 48.3%) randomly selected from patients diagnosed with idiopathic PD according to the UK Brain Bank criteria [16]. Patients with vascular parkinsonism and severe metabolic disorders or those who used vitamin supplements were excluded from the study.

For all patients, the duration of the disease, on-going treatments, and dosage and duration of levodopa therapy were recorded. In addition, patients were also examined for the presence of vascular risk factors, such as hypertension, diabetes mellitus, and hyperlipidemia and for vascular diseases, such as coronary artery disease and stroke.

The stage of PD and the severity of disease findings were determined by the “modified Hoehn and Yahr (mHY) Staging Scale” and “Unified Parkinson's Disease Rating Scale” (UPDRS) II and III, respectively [17, 18]. The dominant motor features of PD were determined by using the subscores calculated from questions about tremor (20, 21), rigidity (22), bradykinesia (23, 24, 25, 26, 31), and balance/postural instability (27, 28, 29, and 30) in the UPDRS III. In addition, the presence of dyskinesia was also noted.

The assessment of global cognitive status was conducted with the standardised “Mini-Mental State Examination” (MMSE). We also noted the presence of visual hallucinations.

Blood samples were collected from peripheral veins into vacuum tubes containing EDTA in the early morning after 12 hours of fasting and 12-hour drug-free periods. Plasma Hcy levels were measured by high-performance liquid chromatography with fluorescence detection (HPLC-FLD). Serum vitamin B12 and folic acid levels were measured by an immunoassay.

The carotid arteries of patients and control subjects were investigated using carotid colour Doppler ultrasonography with a 6 to 11 MHz linear probe performed by the same physician in the Radiology Department who was blinded from the clinical history of 50 subjects. The common carotid arteries and extracranial internal carotid arteries were investigated on the axial and longitudinal planes using the B mode and colour Doppler methods, starting at the origin and including the bifurcation point; intimal thickening, plaques, and stenosis were noted. The frequency levels, gain adjustments, and grey scale and Doppler parameters were manually adjusted to optimise visualisation of stenosis [19].

Among 45 patients, ischemic lesions at the lacunar and specific arterial irrigation areas were recorded by brain computed tomography (CT) and magnetic resonance imaging (MRI) examinations.

The study was carried out according to the Helsinki Declaration and was approved by the Institutional Ethics Committee.

### 2.2. Statistical Analysis

All statistical data analyses were performed with SPSS Statistics (version 19, IBM, Chicago, IL, USA). Prior to the analysis, the normality of the distribution of Hcy levels was examined and the distribution of the 60 values was found to be consistent with the normal distribution (Kolmogorov-Smirnov: 0.066, *p* < 0.05). All analyses were conducted using parametric tests on the data from 60 patients. We established the necessary hypotheses to examine the differences between the patients with different clinical manifestations of plasma Hcy levels and to identify independent variables that affect plasma Hcy levels and tested these hypotheses using* t*-tests, ANOVAs, and correlation analyses.

## 3. Results

The mean age of 60 patients (29 males, 48.3%) with idiopathic PD was 68.4 ± 8.95 years. Demographic and clinical characteristics and serum vitamin B12 and folic acid levels of the patients are shown in Table 1.

In male and female patients, the mean plasma Hcy levels were 16.26 ± 4.57 *μ*mol/L and 11 ± 3.76 *μ*mol/L, respectively. Plasma Hcy levels were significantly higher in male patients (*p* < 0.001). In addition, there was a statistically significant positive relationship between plasma Hcy levels and the age of the patients (*p* < 0.04), whereas an inverse relationship was found between plasma Hcy levels and serum levels of vitamin B12 (*p* < 0.01). There was no significant correlation between plasma Hcy levels and the duration of PD, disease stage, severity of motor symptoms as determined by UPDRS II and III, clinical features, and cognitive status as assessed by standardised MMSE (Table 2).

There was also no significant correlation between plasma Hcy levels and the presence of dyskinesia. Likewise, although the patients with hallucinations had slightly higher plasma Hcy levels, this difference was not statistically significant.

We found no significant correlation between plasma Hcy levels and diabetes mellitus, hypertension, hyperlipidemia, or coronary artery disease. Two patients had a history of strokes; their plasma Hcy levels were 15.5 and 20.4 *μ*mol/L. Although these patients had relatively slightly higher plasma Hcy levels than the mean, this was not statistically significant.

The mean plasma Hcy levels of the patients with intima-media thickening (IMT), atherosclerotic plaques, and stenosis of greater than 50% as detected by colour Doppler ultrasonography of the carotid artery were found to be slightly higher than those of patients without these symptoms, but the difference was not statistically significant. Similarly, we found no significant relationship between plasma Hcy levels and multiple ischemic lesions detected by brain imaging studies (Table 3).

When the correlation between ongoing treatments and plasma Hcy levels was examined, the patients on only dopamine agonist therapy and on LD therapy without COMT inhibitors were found to have the lowest and highest plasma Hcy levels, respectively. However, the difference between plasma Hcy levels and treatment groups was not statistically significant.

Although the dose and duration of LD therapy in patients with plasma Hcy levels >14 *μ*mol/L were slightly higher, this difference was not statistically significant (Table 4).

## 4. Discussion

In PD, motor symptoms occur as a result of dopaminergic cell loss and therefore LD replacement therapy is the most effective treatment option. Patients treated with LD have been reported to have higher plasma Hcy levels [12, 13, 20]. In addition, plasma Hcy levels were higher in PD patients using LD therapy than in patients not using LD and the control group [12, 21]. This increase in Hcy levels has largely been attributed to chronic LD use and, specifically, drug catabolism [21]. LD is subjected to O-methylation via the enzyme COMT in the brain and peripheral tissues. This reaction requires S-adenosylmethionine (SAM) as a methyl donor and, ultimately, yields methylated S-adenosylhomocysteine (SAH). SAH is hydrolysed rapidly and is eventually converted to Hcy [4]. Therefore, long-term LD treatment leads to HHcy by increasing the production of Hcy [4].

Plasma Hcy levels may also be affected by the patient's methylene tetrahydrofolate reductase (MTHFR) enzyme genotype and vitamin B status, except in cases of LD therapy [12]. The MTHFR enzyme needs folic acid and vitamin B12 to function properly. Plasma Hcy levels increase as a result of folic acid, vitamin B6, and vitamin B12 deficiencies and mutations of the enzyme encoding the MTHFR gene [21].

In elderly individuals, Hcy is an independent risk factor for vascular diseases, cognitive deterioration, and dementia [21].* In vitro* studies have indicated a relationship between Hcy and DNA damage, apoptosis, excitotoxicity, and oxidative stress, which are of great importance in neurodegeneration [21, 22]. Neuronal degeneration in peripheral nerves as well as central nervous system during LD treatment in PD patients may also occur due to secondary metabolic changes such as HHcy and low vitamin values of folic acid and B12 [23].

Some studies have suggested a correlation between HHcy, advanced age, and male gender, but this correlation has not been demonstrated in other studies [11, 15, 21, 24]. In our study, we found a positive correlation between plasma Hcy levels, advanced age, and male gender and a negative correlation between plasma Hcy levels and serum levels of vitamin B12. In patients with HHcy on LD treatment, vitamin B12 levels may be lower or within the normal range [15]. As Hcy is generated through demethylation of methionine and hydrolisation of SAH, accumulation of Hcy inhibits methyltransferases through negative feedback mechanisms. Therefore elevated plasma Hcy is associated with reduced methylation capacity. Folic acid and vitamin B12 reduce Hcy levels by reversible metabolism of Hcy to methionine. Negative correlation between plasma Hcy and vitamin B12 levels may reflect the methyl group consuming side effect of chronic levodopa metabolism. Vitamin supplementation decreases Hcy generation by reversible degradation of Hcy to methionine [25].

In our study, as reported in the literature, we did not find a significant correlation between plasma Hcy levels and the duration of PD [13, 15, 24], whereas some authors have noted such a relationship [11]. Although plasma Hcy levels of patients using LD are generally higher than controls, the results of studies investigating the effect of the dose and duration of LD on plasma Hcy levels can be confusing. Some researchers have suggested a correlation between daily dose of LD and plasma Hcy levels, whereas others have suggested no correlation [11–13, 15, 22, 24, 26]. Likewise, there are also studies that showed or did not show a correlation between the duration of LD and plasma Hcy levels [11, 12, 15, 22, 26]. We think that the differences between these results are due to the patient selection criteria, study design, and sample size. In our study, we did not find a relationship between plasma Hcy levels and the daily dose or the duration of LD. Although we did not find a statistically significant correlation between LD dose and plasma Hcy levels, we noted that the plasma Hcy levels of patients on LD therapy tended to be higher. Furthermore, we found no relationship between plasma Hcy levels, the mHY stage of PD, and UPDRS III parameters of disease severity, including the clinical features of the disease, tremor, bradykinesia, rigidity, and gait/postural instability subscores. Absence of correlation between plasma Hcy levels and clinical outcome scores may be explained with the effect of LD treatment on clinical picture. Moreover response to LD may be different in each patient due to the impact of body weight on LD plasma appearance. Therefore, clinical outcome parameters may not always be consistent with the plasma Hcy values in clinical practice.

Following the* in vitro* and* in vivo* observations on the toxic effects of Hcy on dopaminergic neurons in the substantia nigra, some authors have suggested that HHcy associated with LD may play a role in the progression of PD and the development of motor complications and that dyskinesias and motor fluctuations may be due to the toxic effects of Hcy [7, 12].

In one study, the plasma Hcy levels of patients with dyskinesia were found to be higher, but no correlation was found between plasma Hcy levels and the duration of dyskinesia. Hence, it was suggested that plasma HHcy may play a role in the development of dyskinesias due to the toxic effects of Hcy on dopaminergic and nondopaminergic neurons [7].

Hcy is thought to affect dopamine turnover and cause dyskinesia by disrupting the balance of striatal activity. However, some studies have not demonstrated an association between plasma Hcy levels and an increased risk of dyskinesia [12]. In our study, we found no significant difference between plasma Hcy levels of patients with and without dyskinesia.

HHcy is a major risk factor for vascular diseases and has been associated with stroke, myocardial infarction, and dementia in elderly individuals without PD [1–3, 27]. Some reports indicated that LD-induced HHcy in PD patients can lead to atherosclerosis [27]. In screening early atherosclerotic changes, intima-media thickening (IMT) is an easily measured biomarker, which in addition to showing local changes in blood vessels also provides information about generalised atherosclerosis [27, 28].

Previous studies evaluated IMT in patients with PD who were treated with long-term LD therapy and identified hypertrophic changes. Furthermore, these studies found a correlation between the duration of LD therapy and the degree of the intima-media hypertrophy [27].

In the literature, there are studies indicating no correlation between LD-related HHcy and IMT of the carotid artery or even showing a lower IMT in PD patients than in controls [11, 29]. Therefore, the patients with PD are thought to have a lower risk of preclinical atherosclerosis [29]. The autonomic dysfunction and/or hypotensive effects of LD in PD are thought to be protective against atherosclerosis [29, 30].

In our study, we compared the plasma Hcy levels of patients with and without vascular changes, including carotid artery IMT, atherosclerotic plaques, and stenosis, but we did not find any significant correlations.

The prevalence of coronary artery disease has been reported to be increased in patients with high levels of Hcy on LD therapy [31]. However, another study showed no correlation between Hcy levels and carotid artery IMT and cardiovascular morbidity [11].

Ischemic brain lesions may affect the clinical condition in PD. Nonetheless, clinically evident cerebrovascular disease is rarely seen in PD patients [28]. Because of the high incidence of cerebrovascular diseases, it has been suggested that cerebrovascular diseases can coincidentally be seen in some patients with PD [28]. In our patient group, we did not detect a correlation between the plasma Hcy levels of those with and without coronary artery disease. The mean plasma Hcy levels of two patients with a history of stroke were higher than the group mean, but this difference was not statistically significant due to the low number of patients. We suggest that an investigation should be carried out in a larger patient cohort to determine whether these higher plasma Hcy levels were the result of the LD treatment predisposing patients to stroke.

The decrease in blood pressure in PD has been thought to provide protection against stroke [30]. However, if LD-induced HHcy was the only factor that causes vascular complications, there should be data indicating that Hcy-related diseases are more common in patients with PD. However, there is still no convincing data indicating a higher incidence of cardiovascular disease and stroke in PD patients than in controls [11].

Patients with PD have a 4–6 times greater risk of developing dementia compared with the age-matched population [13, 30]. LD therapy has been thought to affect the risk of dementia and cognitive impairment because it increases Hcy levels [20, 26]. Furthermore, the correlation between HHcy and Alzheimer's disease suggests that PD patients with HHcy may be prone to dementia [30].

Dementia is a common nonmotor feature in PD. Moreover, the relationship between increased levels of Hcy and dementia in PD has not been fully clarified, although a significant proportion of PD patients develop cognitive impairment [12, 15].

In a rodent study, Lee et al. showed a decrease in dopamine levels and behavioural changes following intraventricular injection of Hcy [32]. On this basis, HHcy has been suggested to play a role in the development of cognitive impairment in PD [15].

*In vitro* studies suggested that Hcy can enhance oxidative stress, induce accumulation of cytosolic calcium, impair DNA repair mechanisms, and cause neuronal damage and apoptotic death by triggering excitotoxicity through stimulation of NMDA and glutamate receptors [33]. Moreover, Hcy can have cytotoxic effects on endothelial and neuronal cultures [24, 33].

Zoccolella et al. found higher Hcy levels in PD patients than in controls and in PD patients with dementia than in patients without dementia and suggested a significant association between high Hcy levels and dementia [20]. In another study, significantly higher plasma Hcy levels were found in PD patients with cognitive impairments than in those without cognitive impairments and the risk of cognitive dysfunction was found to increase with plasma Hcy levels after adjustment for age, gender, and vitamin B status [15]. Similarly, there are imaging studies indicating that white matter changes in the brain may affect cognition in PD along with the vitamin B12, folic acid, and Hcy levels [34]. However, while previous studies have shown that plasma Hcy levels of >14 *μ*mol/L in elderly people without dementia decreased cognitive performance by 25%, a study conducted on two groups of PD patients with plasma Hcy levels greater and lower than 14 *μ*mol/L found no difference in cognitive performance [13].

In many studies, plasma Hcy levels were correlated with cognitive function, dementia, and markers of neurodegeneration in PD patients [14, 24]. However, some studies showed no correlation between plasma Hcy levels and neuropsychiatric symptoms such as depression, cognitive impairment, and psychosis [11]. Rodriguez-Oroz et al. demonstrated no correlation between plasma Hcy levels and cognitive disorders, dementia, silent infarcts, or genetic polymorphisms [35]. Likewise, in another study, no correlation was detected between plasma Hcy levels and hallucinations [11].

The difference between the results of the studies arises from the retrospective nature of some studies, the small number of patients, and the diversity of neuropsychiatric batteries.

Degenerative brain changes associated with disease are the primary cause of cognitive and motor deterioration in PD. However, comorbid hypoperfusion can contribute to the emergence and severity of this cognitive and motor deterioration [28]. Concomitant vascular pathology aggravates the clinical condition in PD [28].

In conclusion, plasma Hcy levels may be elevated for a variety of reasons in PD, and HHcy is a well-known risk factor for vascular disease and dementia in the elderly. Because of the higher prevalence of vascular diseases and increased risk of cognitive impairment throughout the course of PD, PD patients often have coincidental vascular diseases or dementia. In addition, LD therapy is not the only cause of HHcy. Hence, given the results of our study, we conclude that HHcy has no significant effect on clinical status in PD in terms of motor deterioration, vascular disease, and dementia.

## Figures and Tables

**Table 1:** Demographic and clinical characteristics and values of vitamin B12 and folic acid in PD patients^*∗*^.

	PD (*n* = 60)
Age (years)	68.4 ± 8.95
Duration of disease (years)	7.0 ± 5.75
Age of PD onset (years)	61.4 ± 10.92
mHY stage	2.2 ± 0.99
UPDRS-II score	12.1 ± 6.86
UPDRS-III score	17.4 ± 8.37
UPDRS tremor subscore	2.3 ± 1.56
UPDRS rigidity subscore	1.4 ± 0.90
UPDRS bradykinesia subscore	7.1 ± 3.29
UPDRS gait/postural instability subscore	3.4 ± 2.99
MMSE score	24.2 ± 4.21
Vitamin B12 (pg/ml)	344.4 ± 206.54
Folic acid (ng/ml)	9.3 ± 3.74

^*∗*^Data reported as the mean ± SD.

PD: Parkinson's disease; mHY: modified Hoehn and Yahr Staging Scale; UPDRS: Unified Parkinson's Disease Rating Scale; MMSE: Mini-Mental State Examination.

**Table 2:** Correlation between plasma homocysteine levels and demographic and clinical characteristics and serum B12 and folic acid values.

	Homocysteine levels	
	*r*	*p*
Age (years)	0.27	**0.04** ^**∗**^
Duration of disease (years)	0.23	0.07
Age of PD onset (years)	0.14	0.30
mHY stage	−0.10	0.44
UPDRS-II score	0.09	0.48
UPDRS-III score	0.01	0.91
UPDRS tremor subscore	0.11	0.42
UPDRS rigidity subscore	0.18	0.16
UPDRS bradykinesia subscore	−0.01	0.92
UPDRS gait/postural instability subscore	0.02	0.90
MMSE score	0.03	0.85
Vitamin B12 (pg/ml)	−0.32	**0.01** ^**∗**^
Folic acid (ng/ml)	0.06	0.68

^*∗*^Difference is statistically significant (*p* < 0.05).

PD: Parkinson's disease; mHY: modified Hoehn and Yahr Staging Scale; UPDRS: Unified Parkinson's Disease Rating Scale; MMSE: Mini-Mental State Examination.

**Table 3:** Plasma homocysteine levels and their relationships with hallucinations, dyskinesia, vascular risk factors, carotid colour Doppler US, brain MRI findings, and anti-parkinsonian medication in patients with Parkinson's disease.

		*n*	Homocysteine levels (*µ*mol/L)^*∗*^	*p*
Hallucinations	Absent	46	13.34 ± 4.62	0.13
	Present	14	15.53 ± 4.99	

Dyskinesia	Absent	44	14.14 ± 5.00	0.44
	Present	16	13.05 ± 4.05	

Hypertension	Absent	33	14.15 ± 4.38	0.60
	Present	27	13.49 ± 5.25	

Diabetes mellitus	Absent	51	14.15 ± 4.83	0.24
	Present	9	12.12 ± 4.18	

Hyperlipidemia	Absent	39	13.35 ± 4.48	0.27
	Present	21	14.77 ± 5.23	

Coronary heart disease	Absent	47	13.6 ± 5.02	0.44
	Present	13	14.77 ± 3.67	

Previous stroke	Absent	58	13.71 ± 4.76	0.22
	Present	2	17.95 ±3.47	

Carotid colour Doppler US	Normal	14	12.96 ± 4.18	0.42
	Atherosclerotic findings^†^	36	14.24 ± 5.27	

Brain MRI	Normal	22	13.08 ± 4.88	0.85
	Ischemic lesions^‡^	23	13.37 ± 5.03	

Anti-parkinsonian medication	LD ± DA	31	15.06 ± 4.78	0.07
	LD ± DA ± COMTI	18	12.89 ± 5.08	
	DA	11	11.25 ± 3.26	

^*∗*^Data reported as the mean ± SD.

^†^Atherosclerotic findings including intima-media thickness, atherosclerotic plaques, and stenosis.

^‡^Ischemic lesions including multiple lacunar and vascular territorial infarcts.

US: ultrasonography; MRI: magnetic resonance imaging; LD: levodopa; DA: dopamine agonists; COMTI: catechol O-methyl transferase inhibitors.

**Table 4:** Comparison of daily levodopa dose and duration of levodopa medication between patients with plasma homocysteine levels ≤14 and >14 *µ*mol/L.

	Homocysteine levels (*µ*mol/L)^*∗*^		*p*
	Homocysteine ≤ 14 (*n* = 18)	Homocysteine > 14 (*n* = 31)	
Levodopa dose (mg/daily)	370.50 ± 138.20	430.65 ± 232.26	0.26
Duration of levodopa medication (years)	5.54 ± 5.68	7.65 ± 5.32	0.16

^*∗*^Data reported as the mean ± SD.
